# Lung Cancer and Eye Metastases

**Published:** 2014

**Authors:** Sofia Lampaki, Ioannis Kioumis, Georgia Pitsiou, George Lazaridis, Konstantinos Syrigos, Georgia Trakada, Stylianos Kakolyris, Konstantinos Zarogoulidis, Ioannis Mpoukovinas, Aggeliki Rapti, Paul Zarogoulidis

**Affiliations:** 1 Pulmonary Deparment-Oncology Unit, ``G. Papanikolaou`` General Hospital, Aristotle University of Thessaloniki, Thessaloniki, Greece; 2 Oncology Department, ``G. Papageorgiou`` Hospital, Thessaloniki, Greece; 3 Oncology Department, ``Sotiria`` Hospital of Chest Diseases, University of Athens, Athens, Greece; 4 Department of Clinical Therapeutics, Division of Pneumonology, Medical School, National University of Athens, Athens, Greece; 5 Oncology Department, University General Hospital of Alexandroupolis, Democritus University of Thrace, Alexandroupolis, Greece; 6 Pulmonary Deparment-Oncology Unit, ``Sotiria`` Hospital of Chest Diseases, Athens, Greece; 7 Oncology Department, Biomedicine Private Laboratory, Thessaloniki, Greece

**Keywords:** Exophalmos, Metastases, NSCLC, SCLC

## Abstract

It has been observed that lung cancer either non-small cell or small cell is responsible for eye metastases. This form of metastases in several cases was the first manifestation of the disease and further investigation led to the diagnosis of the underlying malignancy. Both types of lung cancer are equally responsible for this demonstration. Furthermore; both chemotherapy and tyrosine kinase inhibitors have shown equal positive results in treating the exophalmos manifestation. Up to date information will be presented in our current work.

## INTRODUCTION

The metastatic carcinoma to the eye was considered a rare occurrence. Perls reported the first case of choroidal metastasis in 1872 (1). Malignant tumors from other parts of the body may spread to the eye and in the eye. The majorities of intraocular metastatic tumors involve the choroid, but such lesions also invade the ciliary body, the iris, the neural retina, the optic nerve, and in some cases the vitreous. Eighty percent of patients present with a single tumor in only one eye and 20% have multiple tumors, bilateral tumors, or both. Breast and lung cancers represent more than two-thirds of the primary cancer sites (2). In women, cancer metastases that appear in and around the eye usually arise from a breast cancer, and in men from lung cancer. Less common sites of origin may include the prostate, the thyroid, the gastrointestinal tract and the kidney. Lymphomas sometimes also invade eye or adjacent structures. Treatment of these tumors is efficient in terms of both local tumor control and preservation of vision. However, metastatic lesion to the eye is a poor prognostic sign for the long-term survival.

## EPIDEMIOLOGY AND PATHOGENESIS

The most common intraocular malignant neoplasm is the metastatic carcinoma to the eye. Statistics for the US indicate that 20–25% of all deaths are assignable to the cancer (3). Moreover, at the time of death, 1–2.5% of all people have metastatic carcinoma in at least one eye (4). Of these patients, about 10% have one or more metastatic intraocular lesions before death. Nevertheless, approximately 25% of individuals who are diagnosed with metastatic carcinoma of the eye develop that condition as the initial manifestation of their cancer (5).

## OCULAR MANIFESTATIONS

**Figure 1:**
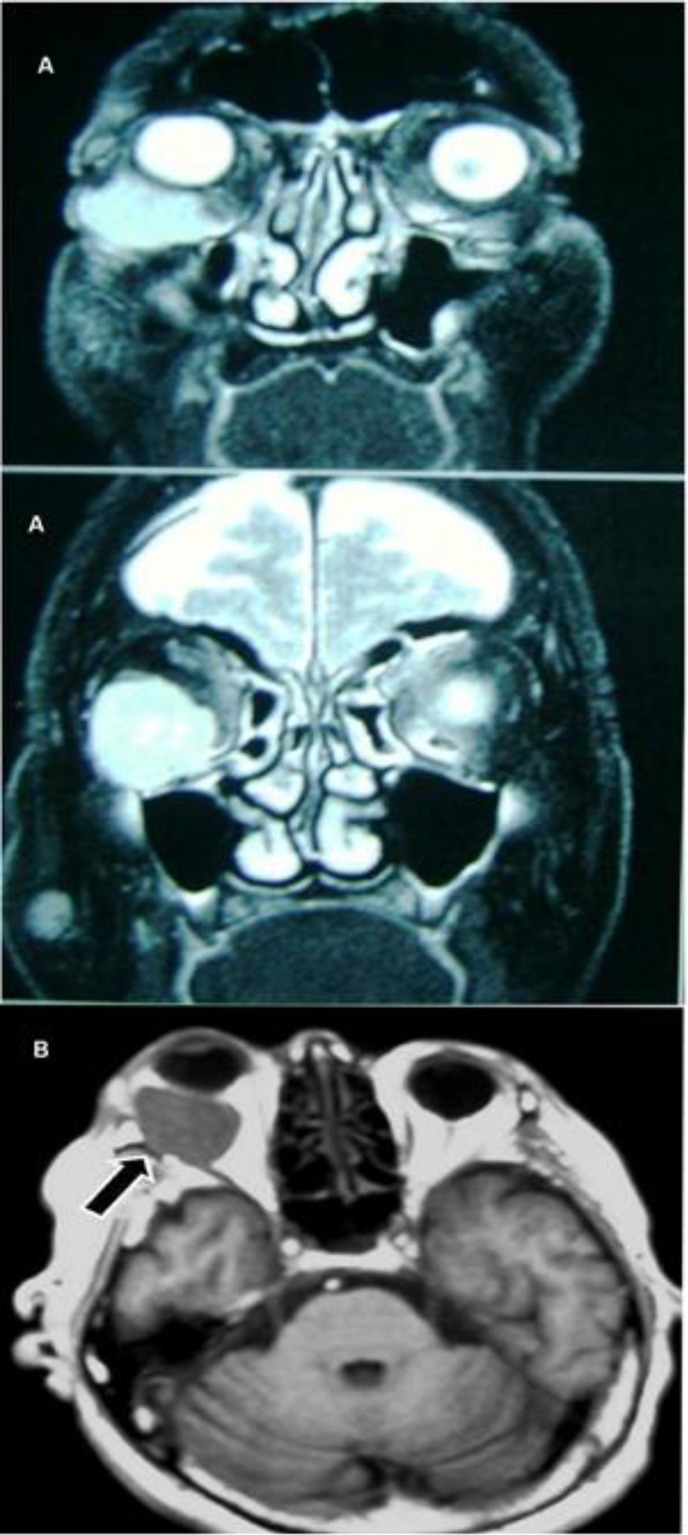
Α) Exophthalmos of the Right Eye, and Β) MRI (Black Arrow Indicates the Lesion).

The principal indication of metastatic carcinoma is blurred or distorted vision in one or both eyes. Usually, the pain is not a sign of metastatic cancer to the eye, except cases that patients have an extensive intraocular tumor. The characteristic metastatic carcinoma to the choroid from lung shows as a golden yellow to yellowish-white round to oval lesion (6). Metastatic carcinoma to the optic disk may appear as a swollen disk without a distinct mass or as a discohesive cellular infiltration of the superficial aspects of the optic disk (7). Metastatic carcinoma to the iris shows as a solid and amelanotic mass (8). Ciliary body metastatic carcinoma appears as diffuse or as multinodular mass, often associated with extensive retinal detachment and severe ocular pain, occurring sporadically. The precise mechanism for the pain in this sort of ocular metastatic carcinoma is not clear (8). Other metastatic carcinomas to the eye include infiltrative lesions of the neural retina (8) and dispersed cells in the vitreous (9). An ophthalmologist who is consulted because of the visual symptoms often diagnoses metastatic carcinomas to the intraocular tissues (Fig. 1).

## DIAGNOSIS

Imaging plays a pivotal role in the diagnosis and treatment of metastatic lesions. Slit-lamp photography is used to document anterior segment tumors. Fundus photography with angiography can detect small or hidden multifocal tumors. Orbital tumors are assessed using CT and/or MRI. Total body PET/CT imaging can be used for systemic staging or to scan occult primary cancers.

Fine-needle aspiration biopsy is a reliable and safe technique for diagnosis in some patients (10, 11).

## MANAGEMENT

The management of patients with metastasis to the eye involves cooperation between the eye cancer specialist, medical oncologist, and the radiation therapist.

The available options for the therapy of ocular metastasis are observation, chemotherapy, photocoagulation, cryosurgery, surgical resection, or radiotherapy. The chosen therapy depends on the clinical condition of the patient. Patient with asymptomatic metastasis close to death probably does not need a therapy. However, a symptomatic patient with controlled systemic disease may be treated to prevent further deterioration in vision (12).

## RADIATION THERAPY

Because lung cancer is radiation sensitive, most patients with uveal metastasis may be treated with relatively low-doses of external beam radiation therapy. In some cases, higher doses of ophthalmic plaque irradiation or enucleation for radiation resistant tumors are required (13).

Radiation incapacitates cells by slicing their DNA. Even more, it destroys blood vessels within and around the tumor. The radiation creates free radicals that combine and destroy cell components, but can affect the normal tissues. Modern radiation is performed either by inserting radioactive elements what is called brachytherapy. Another method is external beam radiation therapy (EBRT). In this case, the radiation is delivered from the outside of the patient and travels through normal tissues to the tumor. Examples of EBRT are linear accelerator (LINAC), gamma knife, electron beam and proton therapy. There are many publications in which the ability of EBRT to treat ocular metastasis successfully and effectively is documented (14–28).

Due to the current improvements in long-term survival of these patients, the incidence of secondary radiation retinopathy and local tumor recurrence has increased. Therefore, a need to decrease the radiation dose exists in order to avoid radiation oculopathy and, on the other hand, late tumor recurrence. The eye and orbit include many normal tissues with varying radiation tolerances. It is possible to lose the eyelashes at least temporarily with low doses or permanently with high-dose radiation therapy. The lacrimal apparatus can tolerate only low-doses, as well. The cornea and scleral eye wall are resistant but the lens is very sensitive to radiation.

## CHEMOTHERAPY

Systemic chemotherapy is effective in treating ocular metastases, especially in chemosensitive tumors such as smal-cell lung cancer. There are many case reports that have shown that chemotherapy is efficient for choroid metastases from non-small-cell lung cancer (29-33).

By a systematic review of the English-language literature, in , of the previously reported patients with choroidal metastasis (most common intraocular malignancy), we found that the majority of patients were male (68%) and were ex- or current smokers (82.3%). The mean age was 54.2 years. The most common histologic type was adenocarcinoma (n = 26), followed by squamous cell carcinoma (n = 12) and small-cell carcinoma (n = 10). The most frequent location of metastatic lesion was the left eye (n = 35), than the right eye (n = 21) or to both eyes (n = 5). Among the patients for whom the location of the primary lesion was specified, the left upper lobe (n = 16) was the most common site. The most common diagnostic modalities were bronchoscopy followed by the lung biopsy (n = 17) and enucleation (n = 15). Liver was the most common extraocular metastatic site identified (35%). Systemic chemotherapy was administrated in 60% of the patients, and the most common outcome was disease progression. Other ocular treatment modalities included radiation (n = 26), enucleation (n = 16), and systemic steroids (n = 9).

Most metastatic carcinomas of the eye are progressive if untreated. Choroidal tumors give rise to bullous retinal detachment, and they can cause anterior shift of the lens and iris. This situation leads to secondary angle-closure glaucoma. Optic disk metastases produce rapid and thorough visual loss. Iris metastases often cause secondary open-angle glaucoma when the trabecular meshwork becomes clogged with tumor cells. If the patients have prolonged survival, most of them, are driven to a blind and painful condition.

## CONCLUSIONS

Metastatic tumors are the most common intraocular malignancies, usually located in the choroid.. The most frequent origins of choroidal metastasis in decreasing order in women are the breast, the lung, the unknown site, the gastrointestinal tract, the skin melanoma. In males the list is headed by the lung, followedthe unknown location, the gastrointestinal tract, the prostate, the kidney and the skin melanoma in men.

The treatment options that are available are the EBRT and the plaque radiotherapy, the surgical resection, the transpupillary thermotherapy and the intravitreal chemotherapy. The use of chemotherapy alone for ocular metastases is not widely reported. All three options of therapy promise. For lung cancer patients with metastatic carcinoma into the eye, there is no a danger to the survival, because the eye is not a vital structure. The prognosis for a patient depends on the response of the lung carcinoma to radiotherapy and/or to the chemotherapy.

Intraocular metastases are significant and under-recognized clinical problem. The overall frequency of ocular metastasis in patients dying of cancer is approximately 16%, and of lung cancer rises about 40%. Careful ophthalmologic examination can make a diagnosis. Radiotherapy is the primary management and allows the most of the patients to maintain useful vision.

## References

[1] Perls M (1872). Contributions to pathology of tumors. Virchows Arch Pathol Anat.

[2] Shields CL, Shields JA, Gross NE, Schwartz GP, Lally SE (1997). Survey of 520 eyes with uveal metastases. Ophthalmology.

[3] Parker SL, Tong T, Bolden S, Wingo PA (1997). Cancer statistics, 1997. CA Cancer J Clin.

[4] Nelson CG, Hertzberg BS, Klintworth GK (1983). A histopathologic study of 716 unselected eyes in patients with cancer at the time of death. Am J Ophthalmol.

[5] Zarogoulidis P1, Terzi E, Kouliatsis G, Androuli S, Kontakiotis T, Zaramboucas T, Zarogoulidis K (2011). Orbital metastases as the first manifestation of lung adenocarcinoma. Case Rep Ophthalmol.

[6] de Bustros S, Augsburger JJ, Shields JA, Shakin EP, Pryor CC 2nd (1985). Intraocular metastases from cutaneous malignant melanoma. Arch Ophthalmol.

[7] Allaire GS, Corriveau C, Arbour JD (1995). Metastasis to the optic nerve: clinicopathological correlation. Can J Ophthalmol.

[8] Shields JA, Shields CL, Kiratli H, de Potter P (1995). Metastatic tumors to the iris in 40 patients. Am J Ophthalmol.

[9] Spraul CW, Martin DF, Hagler WS, Grossniklaus HE (1996). Cytology of metastatic cutaneous melanoma to the vitreous and retina. Retina.

[10] Scholz R, Green WR, Baranano EC, Erozan YS, Montgomery BJ (1983). Metastatic carcinoma to the iris Diagnosis by aqueous paracentesis and response to irradiation and chemotherapy. Ophthalmology.

[11] Augsburger JJ (1988). Fine needle aspiration biopsy of suspected metastatic cancers to the posterior uvea. Trans Am Ophthalmol Soc.

[12] Finger PT (2009). Radiation Therapy for Orbital Tumors: Concepts, current use and ophthalmic radiation side effects. Surv Ophthalmol.

[13] Finger PT, Chin KJ (2012). Antivascular endothelial growth factor bevacizumab for radiation optic neuropathy: secondary to plaque radiotherapy. International Journal of Radiation Oncology* Biology* Physics.

[14] Brady LW, Shields JA, Augsburger JJ, Day JL (1982). Malignant intraocular tumors. Cancer.

[15] Chu FC, Huh SH, Nisce LZ, Simpson LD (1977). Radiation therapy of choroid metastasis from breast cancer. Int J Radiat Oncol Biol Phys.

[16] Dobrowsky W (1988). Treatment of choroid metastases. Br J Radiol.

[17] Hoogenhout J, Brink HM, Verbeek AM, van Gasteren JJ, Beex LV (1989). Radiotherapy of choroidal metastases. Strahlenther Onkol.

[18] Jaeger EA, Frayer WC, Southard ME, Kramer S (1971). Effect of radiation therapy on metastatic choroidal tumors. Trans Am Acad Ophthalmol Otolaryngol.

[19] Tkocz HJ, Hoffmann S, Schnabel K, Niewald M, Ruprecht KW, Schmidt W, Mink D (1997). Bilateral radiotherapy in cases of one-sided choroidal metastases. Front Radiat Ther Oncol.

[20] Maor M, Chan RC, Young SE (1977). Radiotherapy of choroidal metastases: breast cancer as primary site. Cancer.

[21] Minatel E, Trovò MG, Forner L, Franchin G, de Paoli A, Roncadin M, Gobitti C, Bassignano G (1993). The efficacy of radiotherapy in the treatment of intraocular metastases. Br J Radiol.

[22] Nylén U, Kock E, Lax I, Lundell G, af Trampe E, Wilking N (1994). Standardized precision radiotherapy in choroidal metastases. Acta Oncol.

[23] Orenstein MM, Anderson DP, Stein JJ (1972). Choroid metastasis. Cancer.

[24] Ratanatharathorn V, Powers WE, Grimm J, Steverson N, Han I, Ahmad K, Lattin PB (1991). Eye metastasis from carcinoma of the breast: diagnosis, radiation treatment and results. Cancer Treat Rev.

[25] Reddy S, Saxena VS, Hendrickson F, Deutsch W (1981). Malignant metastatic disease of the eye: management of an uncommon complication. Cancer.

[26] Rudoler SB1, Shields CL, Corn BW, De Potter P, Hyslop T, Curran WJ Jr, Shields JA (1997). Functional vision is improved in the majority of patients treatment with external-beam radiotherapy for choroid metastases: a multivariate analysis of 188 patients. J Clin Oncol.

[27] Thatcher N, Thomas PR (1975). Choroidal metastases from breast carcinoma: a survey of 42 patients and the use of radiation therapy. Clin Radiol.

[28] Wiegel T, Bornfeld N, Kreusel KM, Guttenberger R, Hinkelbein W (1997). Radiotherapy for choroidal metastases: interim analysis of a prospective study of the ARO (ARO 95-08). Front Radiat Ther Oncol.

[29] Battikh MH, Ben Yahia S, Ben Sayah MM, Maatallah A, Joobeur S, Rouatbi N, Khairallah M, El Kamel A (2004). Choroid metastases revealing pulmonary adenocarcioma resolved with chemotherapy. Rev Pneumol Clin.

[30] Shields JA1, Perez N, Shields CL, Foxman S, Foxman B (2002). Simultaneous choroidal and brain metastasis as initial manifestations of lung cancer. Ophthalmic Surg Lasers.

[31] Konoglou M1, Zarogoulidis P, Porpodis K, Androudi S, Papakosta D, Matthaios D, Kontakiotis T, Zervas V, Kalaitzidou E, Mitrakas A, Touzopoulos P, Zarogoulidis K (2011). Exophthalmos as a first manifestation of small cell lung cancer: a long-term follow-up. Case Rep Ophthalmol.

[32] Moss HM (1962). Expanding lesions of the orbit A clinical study of 230 consecutive cases. Am J Ophthalmol.

[33] Ahmad SM, Esmaeli B (2007). Metastatic tumors of the orbit and ocular adnexa. Curr Opin Ophthalmol.

